# Generating demand and community support for sexual and reproductive health services for young people: A review of the Literature and Programs

**DOI:** 10.1186/1742-4755-7-25

**Published:** 2010-09-24

**Authors:** Amy J Kesterton, Meena Cabral de Mello

**Affiliations:** 1 Centre for Population Studies, London School of Hygiene and Tropical Medicine, 50 Bedford Square, London, WC1B 3DP, UK; 2 World Health Organization, 20 Avenue Appia, 1211 Geneva, Switzerland

## Abstract

**Background:**

This review investigates the effectiveness of interventions aimed at generating demand for and use of sexual and reproductive health (SRH) services by young people; and interventions aimed at generating wider community support for their use.

**Methods:**

Reports and publications were found in the peer-reviewed and grey literature through academic search engines; web searches; the bibliographies of known conference proceedings and papers; and consultation with experts. The studies were reviewed against a set of inclusion criteria and those that met these were explored in more depth.

**Results:**

The evidence-base for interventions aimed at both generating demand and community support for SRH services for young people was found under-developed and many available studies do not provide strong evidence. However, the potential of several methods to increase youth uptake has been demonstrated, this includes the linking of school education programs with youth friendly services, life skills approaches and social marketing and franchising. There is also evidence that the involvement of key community gatekeepers such as parents and religious leaders is vital to generating wider community support. In general a combined multi-component approach seems most promising with several success stories to build on.

**Conclusions:**

Many areas for further research have been highlighted and there is a great need for more rigorous evaluation of programmes in this area. In particular, further evaluation of individual components within a multi-component approach is needed to elucidate the most effective interventions.

## Background

Approximately 85% of the world's young people live in developing countries where poverty levels remain high and resources are constrained Most will become sexually active before their 20th birthday. In this grouprates of early and unplanned pregnancies, unsafe abortions, maternal deaths and injuries, and sexually transmitted infections (STIs), including the human immunodeficiency virus (HIV) and the acquired immunodeficiency syndrome (AIDS) are very high. It is estimated that more than half of all new HIV infections are among young people, while between one quarter and one half of adolescent girls become mothers before they turn 18. Adolescent girls are two to five times more likely to die during pregnancy or childbirth than women in their twenties [1].

Young people can be defined as those aged 10-24 years; and this group combines adolescents - aged 10-19 years - and youth - aged 15-24 years. While age-appropriate educational needs may exist before this period, it is in this age-range that most people begin to actively explore their sexuality and require sexual and reproductive health (SRH) information and services. While needs vary considerably within the age-group, and between young people in very different circumstances (married/unmarried, parents, students, workers etc) the statistics above testify that these are not being met and appropriate, targeted, evidence-based intervention is required. In 2006 the Joint United Nations Program on HIV/AIDS Interagency Task Team on HIV and Young People began to look into this problem, and a major review of available evidence on preventing HIV in young people in developing countries was carried out by WHO. Despite constraints imposed by the quality of the data, it concluded that "if countries want to move towards achieving the global goals on HIV and young people, there is sufficient evidence to support widespread implementation of interventions that include elements of training for service providers and other clinic staff, making improvements to facilities, and informing and mobilizing communities to generate demand and community support" [2].

In response to the recommendation about mobilizing communities to generate demand and community support, and in recognition of the failure of approaches focusing solely on improving youth SRH service provision (quality, availability, acceptability, accessibility) or the supply side, the WHO Department of Child and Adolescent Health and Development (CAH) supported this further review. The aim was to explore the evidence-base for demand side or community-based interventions focusing on the behaviors of the intended recipients of services.

The review looks firstly at interventions seeking to increase young people's demand for SRH services in developing countries and secondly, at those seeking to increase community support for their use. The review sought to include interventions integrating HIV as part of wider SRH. This is in response to the fact that HIV is largely a sexually transmitted disease and because the high levels of funding directed to fighting the epidemic compared to SRH have resulted in disconnected programming on these substantively related technical areas. The results of this two-part review are summarized here in this paper.

## Methods

Studies were identified through searches of the following electronic publication databases:

PsychINFO, AIDSLINE, MEDLINE, POPLINE, ERIC, Sociological Abstracts, Social Sciences- Wilson Web, Leeds Sexual Health Database, Eldis and Id21. The search terms used were: 'adolescent/youth reproductive health seeking', 'adolescent/youth health', 'adolescent/youth/young people & reproductive health', 'adolescent/youth/young people & sexual health', 'adolescent/youth/young people health service utilisation', 'adolescent/youth/young people health service demand', 'adolescent/youth/young people health community intervention', 'adolescent/youth/young people health & community support'.

Additionally the websites of organizations with related programming and research were thoroughly explored, these included Aids Education Information Service (AEGIS), AVERTing HIV and AIDS, Better Care Network, Core Initiative, Center for AIDS prevention studies, Development Gateway, Department for International Development, EUROPEER, Family Health International (FHI), Interagency Youth Working Group (IYWG), International Center for Research on Women (ICRW), United Nations Joint Program on AIDS (UNAIDS), the United Nations Population Fund (UNFPA), United Nations Educational, Scientific and Cultural Organization (UNESCO), United Nations Fund for Children (UNICEF), World Health Organization (WHO), the Centre for Development and Population Activities (CEDPA), Alan Guttmacher Institute, EngenderHealth, Population Council, International Planned Parenthood Federation (IPPF), Marie Stopes International (MSI), Pathfinder International and PATH.

The bibliographies of known conference proceedings, papers and journals with published review articles were also looked at. This included the report of a technical consultation on the role of community involvement in improving reproductive health and preventing HIV among young people led by CARE/YouthNet and Family Health International (FHI) (November 8-9, Arlington, Virginia, USA. 2005) as well as the accompanying literature review which included a summary of concepts, operations, evaluations, challenges, and emerging themes with an extensive bibliography on the subject Finally consultation with experts was used to increase coverage of the grey literature. This included a meeting organized by WHO African Regional Office in Accra in February 2008 whichincluded representatives from International HIV/AIDS Alliance, PATH, Population Council, WHO, and African governments.

The studies found were all then reviewed against the inclusion and exclusion criteria outlined in Table 1 below. These relate to the location of the program (whether they had been conducted in a developing country), the outcomes measured (demand for and increase utilization of health services by adolescents or community support for and acceptance of provision of young people's health services and their use) and evaluation design (interventions using RCT or quasi-experiemental designs and when outcomes measured are particularly relevant, studies using before and after or cross-sectional designs).

**Table 1:** Inclusion and exclusion criteria for the review

Inclusion criteria	Exclusion criteria
**Part 1. Youth demand for SRH services**	

**Location**	
Programs/studies carried out in developing countries with sufficient details of intervention content.	Programs/studies carried out in developing countries with insufficient details of intervention content.

**Outcomes**	
Programs/studies that attempted to generate demand for and increase utilization of health services by young people;	Programs/studies that did not attempt to generate demand for and increase utilization of health services by young people;

**Evaluation design**	
Intervention studies using the following designs:	Interventions that did not use designs enabling the reader to evaluate the impact of the intervention or to make inferences based on statistical tests.
• randomized controlled trials;	
• quasi-experimental study designs.	
When outcomes measured are particularly relevant, studies using these additional designs were included:	
• data collected before and after (without	
comparison group);	
• cross-sectional (after only) when compared	
with others not exposed to the intervention or	
presented by level of exposure.	

**Part 2. Community support for SRH service use by young people**	

**Location**	
Programs/studies carried out in developing countries with sufficient details of intervention content.	Programs/studies carried out in developing countries with insufficient details of intervention content.

**Outcomes**	
Programs/studies that attempted to generate community support for and acceptance of provision of young people's health services and their use by young people.	Programs/studies that did not attempt to generate community support for and acceptance of provision of adolescent health services and their use by young people.

**Evaluation design**	
Intervention studies using the following designs:	Interventions that did not use designs enabling the reader to evaluate the impact of the intervention or to make inferences based on statistical tests.
• randomized controlled trials;	
• quasi-experimental study designs.	
When outcomes measured are particularly relevant, studies using these additional designs were included:	
• data collected before and after (without	
comparison group);	
• cross-sectional (after only) when compared	
with others not exposed to the intervention or	
presented by level of exposure.	

**Figure 1:**
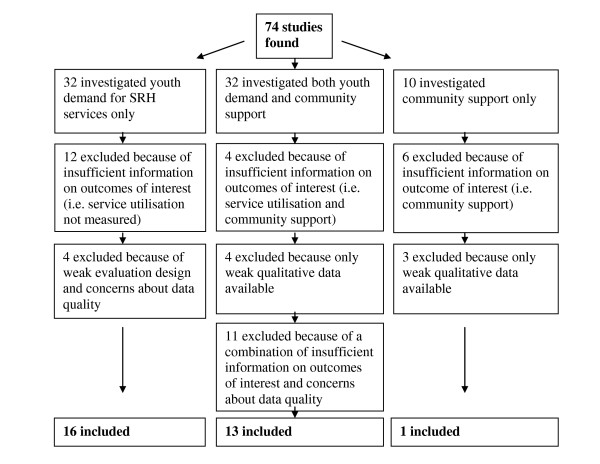
**Flow chart showing exclusion of studies**. Chart shows how studies were excluded from the review due to insufficient information on outcomes of interest, weak study design and issues with data quality.

All the studies found took place later than 1990 so none were excluded on the basis of timing. However, studies were excluded for other reasons and the flow chart in figure 1 below outlines this.

## Results

A total of 74 studies were found through the range of searches. Thirty two studies investigated to some degree increasing both, young people's demand for SRH services, and community support for their use. A further 32 looked only at youth demand, and 10 focused only on generating wider community support.

After reviewing them against the inclusion and exclusion criteria it was found that in total twenty-nine studies met the inclusion criteria for increasing youth demand for SRH services (Part 1.). Thirteen of these studies also met the inclusion criteria for investigating the generation of community support for service use, and they were combined with one additional study, giving a total of fourteen (Part 2).

The studies were critically reviewed by type of intervention. Interventions were categorized in a way which best captured the variety of approaches. The first are those guided by the intervention setting: school, community, youth centres and outreach from health facilities. The cross-cutting methodologies of peer education and life skills education and media, are then looked at separately as they are commonly used in a wide variety of settings. Finally finance interventions and multi-component approaches were investigated as these do not fit in to the above categories. Studies exploring community support for service use were looked at by approach and included targeted community education sessions, festivals and sporting events, the media and broader community mobilization approaches.

The intervention design and evaluation method for each study has been summarized and the strength of evidence assessed. The information is contained in additional file 1.

### Results Part 1: Interventions to increase youth demand for SRH services

#### In-school education

*In-school interventions *benefit from a ready-made audience and there is reasonably strong evidence of the benefits of using curriculum-based participatory and life skills approaches to increase knowledge and awareness [3]. However, there are few evaluations of impact on utilization of services. Only 6 studies met the inclusion criteria, and they gave mixed results.

Strong evidence for the potential impact of setting up active referral systems between schools and health facilities was found by a Randomised Controlled Trial (RCT) in Nigeria [4] in which multivariate logistic regression found a significant increase in service use and reduction in sexually transmitted disease symptoms as a result of the intervention. In Brazil a similar intervention was implemented but a quasi-experimental evaluation using a matched control contrastingly found no significant impact on service use. However, three potential sources of bias were highlighted, weakening the results. Firstly schools and clinics were chosen in part due to their willingness to participate, secondly "before and after" groups differed due to high drop-out and reasons for drop out were not collected and thirdly logistic regression only controls for chosen variables and there may be other factors correlated with the outcomes of interest [5].

The other studies were from the Population Council Frontiers programs in Bangladesh, Kenya, Mexico and Senegal. These all used a quasi-experimental intervention design to test the effect of in-school education additional to that of community mobilization activities and youth friendly health services. A consistent pattern was not found between the countries. No impact on service use was found in Mexico or Kenya. In Bangladesh service use doubled in the intervention area without the school component, and increased ten-fold when it was included, but no significance testing was carried out. In Senegal a significant increase (p < 0.05) above the control was only found when the school intervention was included. This suggests that effectiveness is highly specific to the context and particular details of intervention implementation and interventions need to be carefully tailored. However, in no country were differences between control and intervention populations controlled for. This means where increases were observed the evidence that this was due to the intervention is weak. In many cases youth SRH activities led by other organisations were going on in both control and intervention areas [6-9].

#### Community-based facilitated education sessions

Community-based sessions have the potentially added advantage of reaching out-of-school young people who are often more vulnerable. Sessions can be more informal and relaxed, promoting greater openness and participatory discussion than in school. Evidence suggests however that sessions within the community may struggle to maintain attendance over a period of time, with other commitments often getting in the way [10]. As with in school education, participatory and life skills approaches show the greatest potential and the use of community members to carry out education in a culturally sensitive way also shows promise, as does the combination of these sessions with wider community mobilization activities [11].

There is evidence of community-based education sessions influencing knowledge of youth SRH issues and some related behaviours [10], but only 2 studies met the inclusion criteria and tested impact on uptake of services. Both studies are from India involve the same organization, International Centre for Research on Women (ICRW) and focused on married young people. The first, in Maharashtra used a "before and after" study to test the effect of community education and counseling sessions. The endline survey showed an increase in service use with 10% of clients referred from education sessions and 30% from counseling. However, the lack of a control and significance testing of the increase in service use weakens the strength of evidence. The other study used a quasi-experimental design comparing the use of social mobilization techniques with the establishment of youth friendly services. Social mobilization techniques include discussions, role plays and other participatory exercises which enable a dialogue among community members and facilitate a decision-making process. In general social mobilization was found more effective than the provision of youth friendly services in increasing service use but only percentage increase was reported without significance testing. In both studies therefore the strength of evidence connecting the intervention to changes in service use is relatively weak [12].

#### Youth centres

Youth centres in the community represent existing structures, much like schools, that provide a ready-made target audience, but they also have the added benefit of involving out-of-school young people. There are many examples of youth centres being set up with a youth SRH purpose in mind, and these tend to combine SRH services with recreational activities to attract young people, as well as providing vocational and educational components. Evidence shows there has been some success in promoting youth centres for information and recreation but in general evidence for encouraging young people to access services is poor. It has also been found that people attracted to youth centres for services tend to be older than the target age and that they are often female [10,13].

Only three studies using youth centres to provide services were found for inclusion in this review. Centre Dushishoze in Rwanda [14], Pathfinder International's centre in Gweru Zimbabwe [15] and the ABTEF youth centre in Togo [16] all combined the provision of services with recreational activities, peer education and in the case of Togo, use of the media. In Rwanda, Zimbabwe and Togo clinic data was reviewed over time but without a control. In Zimbabwe no effect was found. In Togo youth were found more likely to use the youth centre clinic than other clinics in the area and a moderate (non-significant) increase in use over time was observed. However, problems were encountered obtaining the follow-up data i.e. it was biased compared to the representative baseline sample with youth more likely to be male, in-school and more educated, all factors possibly associated with the outcomes of interest. In Rwanda a more sophisticated analysis was possible as level of exposure to the intervention was measured. Change in service use was found significantly related to exposure (p ≤ .05) when age, residential area, level of education, school enrolment were controlled for.

#### Information, Education, Communication (IEC) outreach from health facilities

Outreach and promotion of their services by facilities can involve IEC materials, community and peer educators providing referrals and use of the mass media. These techniques can be used as part of a broader social franchising model, in which providers in the franchise use common marketing and branding. Socialfranchising involves the application of franchising techniques such as promotion, training and quality assurance to social enterprises. It can be used by government, not-for-profit and private sector providers.

Five studies looking at IEC outreach from health facilities have been included in this review. A study from Thailand involved the innovative use of pharmacists to provide information, counseling and referral of young people to services. Government health facilities in the referral network experienced an increase in young service users but while service statistics were collected overtime there was no control to strengthen evidence that the increase was due to the intervention [17].

Two other studies looked at social franchising programs, Top Reseu in Madagascar and Mexfam in Mexico [18]. In these the training of service providers and branding of franchises were combined with activities in the community such as peer education, media and community education sessions. Both showed an increase in service use but as in Thailand, only before/after data was collected.

In Mongolia [19] and China [20] similar outreach activities were carried out but without being part of a franchising model. A more rigorous quasi-experimental evaluation design was used. In Mongolia 10-19 year old males and females were found statistically significantly (p < 0.05) more likely to use services in project than control sites. However, differences in catchment populations were not controlled for. In China while there was a significant increase in contraceptive use and knowledge of available services, uptake was not measured.

#### Peer education and counselling

Peer education is a popular and flexible approach that has been used in many different contexts (e.g. schools, universities, youth clubs and the community). Peer education and counseling programs vary considerably in objectives and operations. All provide education, but peers may also act as counsellors or condom distributors, and they may provide referrals to formal health services. However, the impact of these activities are often not closely monitored. Evidence suggests that peer education is most effective as a component of wider interventions [13]. Educators themselves seem to benefit the most, and although there is evidence of some impact on the knowledge and behaviour of recipients and on condom use, the evidence is weaker for uptake of services [21]. No studies specifically looking at the impact of peer education on demand and utilization of SRH services by youth were found which met the inclusion critieria. However, peer education is a component of many multi-component approaches looked at later.

#### Life skills education or broader youth development approaches

Life skills approaches grew out of the failure of narrow problem-specific education programs and aim to address the wider determinants of youth behavior increasing adolescents' autonomy, mobility, self-esteem and decision-making and placing SRH in the broader context of young peoples' lives [22-24]. Two studies have been included, both from India. The Better Life Options Program (BLP), using a quasi-experimental design found that BLP alumnae were significantly more likely to have been to a health centre alone in the last six months, and to have used prenatal, delivery and postnatal care in their last delivery, than those in the control. However in the analysis, differences between the control and intervention groups were not adjusted for and there was no baseline comparison survey [25]. In the Swaasthya program there was a before/after evaluation, but no control and although there was an increase in knowledge of services, uptake was not measured [12].

#### Use of media

Use of the media covers a wide spectrum of different approaches, from the distribution of printed IEC materials at health centres, schools, workplaces and other locations to comprehensive mass media campaigns using television and radio.

Disentangling the impact of media efforts on knowledge and behaviour is difficult and, in general, evidence suggests that they are more effective in influencing the former. They also seem most effective when several complementary approaches are used [13]. There is only weak evidence that media efforts successfully increase uptake of services, and only two studies met the inclusion criteria. In Zimbabwe, use of the radio, launch events and drama were combined with IEC material distribution and peer education to promote youth friendly services. A quasi-experimental design with 5 intervention areas and 2 controls found young people in campaign areas were significantly more likely to visit a health centre than the control, when differences were adjusted for. However, contamination of the control weakens the evidence [26,27]. The other program, in Burkina Faso, used folk and modern media but it was only a before and after study measuring knowledge of how to obtain services but not service use. Knowledge did increase but no significance testing was carried out [28].

#### Voucher finance interventions

The development of schemes providing vouchers for subsidised or free SRH services to youth with the aim of encouraging uptake is at an early stage. Such schemes are able to utilize the private sector and give adolescents a choice of services so that they can pick the ones that they feel most comfortable with. They do require careful monitoring, however, and may be labour-intensive to run [29]. Two studies met the inclusion criteria. Results from a quasi-experimental study in Managua, Nicaragua are promising with use of services amongst recipients of vouchers found significantly (P < 0.05) higher than amongst non-recipients when differences in the populations were controlled for [30,31]. In Kenya the use of vouchers was integrated into a wider program including peer education and community sensitization. Most of the vouchers were used as recipients were followed up, but no statistical comparison of service use was made between control and intervention area [32].

#### Multi-component and multi-sectoral approaches

Many programs use a combination of interventions to try and influence youth knowledge and behaviour. Seven multi-component studies have been included here but none test the statistical significance of changes in service use and only one study, in Nepal, used a control.

In Youth Now Jamaica, Geração Biz Mozambique and Save the Children's programs in Ethiopia Malawi, Bhutan, Vietnam and Nepal, the use of school and community education, the media and youth friendly services resulted in an increase in service use. However, the before/after evaluations used do not allow the change to be definitively linked to the intervention [33-36].

The African Youth Alliance programs in Botswana, Tanzania, Ghana and Uganda did not include a control either, as the programs were nationwide. However, component specific evaluations were carried out to try and link changes more directly to specific interventions. Service use was reported to have increased, but without detailed data reported, and a focus was placed on analysis of changes in knowledge and contraceptive use [37]. This was also the case in the RHIYA program in Bangladesh, Cambodia, Laos, Nepal, Pakistan, Sri Lanka and Vietnam with detailed service uptake data not available [38,39].

In Nepal the specific effect of community participation in intervention design and implementation was investigated using a quasi-experimental design. In the control traditional peer education, school education and youth friendly services were implemented while in the intervention area community participation meant a greater variety of interventions including youth clubs, livelihoods training and street theatre. Deliveries in medical facilities increased in both sites but were much more substantial in the study site. However, no significance testing was carried out, or adjustment for differences in the study populations, and this weakens the strength of the evidence [40].

### Results Part 2: Interventions to increase community support for SRH service use by youth

#### Community sensitisation via multimedia

Only one study met the inclusion criteria and provided some evidence of a media campaign creating a more supportive environment for youth to access SRH services. In Zimbabwe mass media was combined with peer education, distribution of IEC materials and youth friendly services in a quasi-experimental study. It was concluded that building community support for behavior change is essential to ensure that young people find approval for their actions and have access to services. It was found that multi-media is a good way of doing this and logistic regression showed increased communication on reproductive health reported between siblings, friends and youth and their parents in campaign areas. However, contamination of the control weakens the evidence [26,27].

#### Community participation and mobilization

Community participation encompasses a continuum of approaches from inclusive collective action and mobilization to simple community education and awareness-raising. These approaches are increasingly being used to improve community ownership and involvement in programming. Nine studies met the inclusion criteria and demonstrated some impact on nurturing a supportive environment for youth utilization of SRH services.

In Lusaka, Zambia a quasi-experimental design was used to compare the impact of varying degrees of community mobilization with the improvement of the youth friendliness of services. A positive but non-significant correlation was found between community acceptance of the provision of youth SRH services and their use (outpatient, family planning and reproductive health services). In contrast greater friendliness of services was only associated positively with family planning use. However, small sample size limited statistical power meaning it is likely that significant relationships may have been missed. It was also not possible to control for unobserved differences amongst clinics that may have predisposed some to attract higher levels of clients or unobserved community level factors that may have differentially predisposed youth to using clinic services in some communities [41].

In Jamaica, a before and after study used only qualitative data to evaluate the impact on community acceptance. Findings did show an improvement in negative attitudes towards adolescent sexual activity however, led particularly by religious leaders [33]. Similarly in the Geração Biz program in Mozambique and African Youth Alliance Programs in Ghana, Tanzania, Uganda and Botswana qualitative data demonstrated how community support for implementation of the program was established through communication and mobilization activities [37]. In Mozambique the involvement of parents as community activists was instrumental [34].

In the RHIYA program countries (Bangladesh, Cambodia, Laos, Nepal, Pakistan, Sri Lanka and Vietnam) substantial variation in attitudes towards sexuality and sexual health of young people was found. In Pakistan for example, where sensitivity was high, it was recognized that support at the community level would be necessary for the program to be really effective. Extensive community mobilisation and targeting of key gatekeepers enabled youth centres providing services to be established. Outreach to parents was also found important, for example in Nepal parents were reluctant to educate their children on SRH but the formation of support groups and sensitization of workshops helped them develop the necessary skills [38].

In all the Frontier program sites community acceptance for the provision of reproductive health education and services was reasonably high at the baseline. In Bangladesh this meant change was not measured. In Senegal acceptance was found to vary by topic, with contraceptives the most sensitive, but approval increased during the intervention. In Senegal, Mexico and Kenya, communication of youth with their parents increased significantly in some sites, this included the controls making the impact of the intervention unclear. Differences in the characteristics of the different samples were not adjusted for [6-9].

#### Community participation with involvement in intervention design

Four studies were found which had a particularly strong focus on community participation, and specifically allowed communities to be involved in intervention design. In Nepal a quasi-experimental study design specifically tested the effect of a participatory approach versus implementation of a traditional intervention. Adolescents in the participatory intervention site were found more likely to discuss SRH with their parents, than those in the control, although significance testing was not carried out. Additionally it was found that the participatory approach had a greater effect on community empowerment and capacity building, social norms and other contextual factors that will also influence sustainability Nepal. Detailed changes in adult attitudes were not reported however [39]. In Burkina Faso it was similarly felt that the participatory approach used helped to create an enabling environment which will encourage young people to take charge of their SRH, communication improved but again significance was not tested [28]. Quantitative and qualitative evidence from the two ICRW interventions with married young people in India found the involvement of mother-in laws and husbands in improving young married women's care seeking was found crucial as these are commonly the primary gatekeepers [12].

## Discussion

There are some potential biases in this review that need to be highlighted. The review was limited to programs that have undergone some form of formal evaluation and for which some output data have been compiled and reported. However, these programs represent only a small proportion of the total youth SRH initiatives that have been or are being undertaken in the context of resource poor countries. It is therefore not possible to assert that the interventions included in the review are representative of all interventions in terms of their success in generating demand and community support for the use of youth SRH services. This inadequate level of evaluation and publication of findings is likely to be due to a range of factors. These include insufficient attention from funding agencies to ensure it is part of project design and a lack of training of researchers in appropriate methodologies to evaluate these types of intervention and to meet the publication requirements of international journals.

It is also worth noting that papers included do not comprehensively cover all groups of young people (e.g. young migrant workers or soldiers) or all intervention types (e.g. traditional theatre). They also focus specifically on resource-constrained settings, where need is high and service availability is low, these difficult social and economic environments need acknowledgement. Additionally, although studies in all languages were sought out, all of the studies found and reviewed were in English. It is likely therefore the findings of studies published in other languages are under-represented.

Even amongst those programs that are formally evaluated the review found that relatively few youth SRH programs measure their impact on service use. Most focus on changes in knowledge and if behavior change is included, on contraceptive use. Equally, while community participation in youth SRH programming is widely deemed a good thing, very few studies measure the impact of an intervention on the attitudes of key individuals or groups. Parent-child communication is the main indicator collected quantitatively and qualitative data is not as rich and in-depth as is needed. In those studies that did investigate youth demand or community support for SRH services the quality of the evaluation design is not generally strong, with only one Randomised Controlled Trial found. This meant the inclusion criteria had to be relaxed and made a systematic review impossible. Overall the evidence base for the two areas of demand and community support for young people's SRH services has been found under developed.

There are also some difficulties in generalising review findings, particularly in the multi-country studies. Because information in the evaluations was sometimes missing or insufficient, it was not possible to say whether the interventions that failed to generate significant demand for service use and community support failed to do so because the intervention used was not a good one for that context or because it was not well implemented. Contextual and implementation factors seem to be very important in the effectiveness of the interventions.

### Interventions to increase youth demand for SRH services

In school, the most promise is shown by the potential for setting up formal referral networks between schools and health centres as demonstrated in an RCT by Okonofua et al [4] in Nigeria. Further trials are needed, particularly in places where this may be highly sensitive and involvement of parents and community leaders necessary.

There is also evidence from India to suggest a similar system of referral could be possible from community based education programs reaching out of school youth [12]. Further research is needed into strengthening links to services but also into sustaining education and counseling sessions in the community and ensuring regular participation of youth over the longer term.

In general, the high costs of maintaining youth centres, compared to the costs of supporting outreach/peer promotion components of interventions, does not seem to be justified [42]. However, research to date has focused on uptake of their own services and investigation of the impact of youth centres and their activities on uptake of other services available in the community would be useful.

IEC outreach activities from health facilities show potential for increasing demand, as do the more comprehensive social marketing and social franchising approaches. In particular use of the private sector (e.g. pharmacists) requires further investigation as there is some evidence of young people's preferences for this when seeking commodities and services [17].

Further work is needed to specifically investigate the link between peer education and service use. Preliminary findings from a program in Haryana, India, for example, are beginning to show an impact of peer group educators on visits to health facilities by adolescents. This study is not yet completed and was therefore not available for inclusion in the review [43].

Life skills approaches show potential in a variety of settings including schools and the community, and are certainly more effective than narrow didactic approaches [12,24,25]. Further research is needed however, particularly looking at sustaining changes after the education period.

The use of printed IEC materials may be a valuable educational component, but alone are unlikely to produce changes in behaviour. Mass media is unique in its ability to reach large numbers of people easily, particularly if a variety of mediums are utilized [26-28]. Further work is needed to specifically investigate and disentangle its impact on service uptake.

The provision of vouchers for subsidized or free ASRH services shows promise but there is a lack of rigorously evaluated studies and a need for more research. The schemes have the ability to target adolescents even within a politically conservative context, and of realizing unmet demand for care. Such schemes are able to utilize a range of providers, including the private sector so that youth can pick what they feel most comfortable with. This can however make them financially intensive as well as labour intensive to run [30,31].

Multi-component strategies can reach youth through a variety of complementary channels through school, in the community and at home. These approaches show great potential and more large-scale, innovative, integrated, multifaceted research interventions in youth SRH are needed [36,37,40]. It is important that evaluation allows the impact of individual intervention components on service use to be measured as well as the combined impact of them all together.

### Interventions to increase community support for youth SRH services

Youth are influenced by other individuals, their families, school, and community and societal factors. There is evidence that dealing with youth in isolation is not helpful and that tackling at least some of these areas and influential people in young people's lives may be necessary to sustain changes in behaviour. Overall, it seems that a comprehensive approach is most promising and that programs need to involve the adults around young people(teachers, parents etc) and that broader community mobilization activities can help gather even wider support for youth SRH programs and ease some of the barriers to adolescents accessing services [22].

As mentioned above mass media techniques have the advantage of being able to achieve very wide coverage and also to reach wider community members as well as youth and provide population-level sensitization to youth SRH issues. If sustained, there is the potential to influence social norms and practices - especially if messages are reinforced through other means. However, it is very difficult to directly link changes to a media intervention.

Community and adolescent involvement in the design, implementation and evaluation of interventions can help ensure that they not only meet the needs of the population, but also bring a sense of ownership that helps sustain the interventions in the longer term.

Education sessions that involve the wider community rather than just adolescents have the same problem of sustaining interest and attendance. However, they have the advantage of being able to encourage intergenerational dialogue and wider discussion even on taboo subjects, and they help to break down stigma and discriminatory attitudes. Sessions may be ongoing but are commonly concentrated in the first phase of programs in order to gain support for the implementation of other intervention components, including the provision of services. In some cases it is necessary to make key actors in the community, such as parents and community leaders, an important focus of activities. For example, many parents and community leaders recognize that there is a need for youth SRH education, but they usually do not agree to extensive or intensive education on sex or sexuality. They think adolescents will be promiscuous if they learn about sexuality and contraceptives and do not understand the risk of not providing information [38].

Although the evidence is generally weak, there is some suggestion of the protective effects of positive attitudes of parents and it has been observed that, in some contexts, it is impossible to address youth SRH needs without support from key leaders and constituents. The targeting of key actors, especially at the beginning, is therefore a crucial phase in some interventions. Community IEC activities at festivals, celebrations and sports events, and use of media can reach large numbers of people and have been used to draw attention to SRH activities. There is potential for them to contribute to nurturing community support for addressing youth SRH. However, these events must be part of a wider intervention and must be sustained rather than one-off in order to have a lasting impact.

There is chronic difficulty in disentangling the influences of different interventions within multi-component approaches, and further work is needed to determine the effects of each intervention on knowledge, intent and behaviour. Also, studies do not tend to look at the long-term impact of interventions or at the sustainability of changes.

## Conclusions

The sexual and reproductive health needs of adolescents are severely underserved and the provision of youth-friendly services alone is not sufficient to meet them. Supply side interventions needs to be combined with demand side activities to create a more supportive environment for adolescent care seeking and increased uptake of services, and governments need to work in partnership with civil society and community organizations to reach young people effectively. The intention of this review was to gauge the strength of evidence for different intervention types and on this basis make recommendations for implementation in a variety of resource constrained settings. However, the limited number of studies explicitly measuring impact on service use and community support, and the prevalence of multi-component approaches, making it difficult to disentangle the effects of individual interventions, means this could not be done as rigorously as hoped. The appendix table summarizes the available evidence to inform the potential scaling-up of different intervention types and to guide national policy-makers, program planners and donors in deciding how to allocate the limited resources available for improving young people's SRH and meeting international commitments in this area. The International Conference on Population and Development (ICPD), held in Cairo, Egypt in 1994, called on governments, in collaboration with non-governmental organizations, to meet the special needs of adolescents (ICPD 1994). In 2009, during the 15^th ^Anniversary of ICPD, countries recognized that significantly more work is needed to achieve its goal.

The generally weak evidence base means that all intervention types need further investigation. It is essential that implementation of all interventions should be accompanied by careful monitoring and evaluation, and findings need to be published and made publicly available. Ultimately young people need relevant information, life skills and access to care and these can be provided in a variety of ways [16]. Evidence suggests that effectiveness is highly specific to the context and to particular details of intervention implementation and that interventions need to be carefully tailored [6-9].

## List of Abbreviations Used

The following abbreviations are used in the paper; (AIDS): Acquired Immuno-Deficiency Syndrome; (HIV): Human Immunodeficiency virus; (ICPD): International Conference on Population and Development; (IEC): Information, Education and Communication; (SRH): Sexual and Reproductive Health; (STI): Sexually Transmitted Infections; (WHO): World Health Organization.

## Competing interests

The authors declare that they have no competing interests.

## Authors' contributions

AK carried out the review of literature and programmes and drafted the paper. MCM led in the design of the study, provided guidance to the research and made substantial contributions to drafts of the paper. Both authors read and approved the final manuscript.

## Authors' information

AK has a PhD in public health medicine from the London School of Hygiene and Tropical Medicine and has worked for a number of international NGOs in the field of sexual and reproductive health. MCM is a scientist with considerable experience working for the World Health Organization in the areas of reproductive health, child and adolescent health and development, including mental health.

## Supplementary Material

Additional file 1 **Study summary table**. The summary table provides information on each study's location and dates, target population and objective, evaluation design, intervention description, key findings and effect size. Click here for file
