# Supplementary material for: Generating demand and community support for sexual and reproductive health services for young people: A review of the Literature and Programs
Source: Reprod Health. 2010 Sep 24;7:25. doi: 10.1186/1742-4755-7-25 (PMC2954841; doi:10.1186/1742-4755-7-25)
Supplement: Additional file 1 — Study summary table. The summary table provides information on each study's location and dates, target population and objective, evaluation design, intervention description, key findings and effect size. [file 1742-4755-7-25-S1.DOC]

**Additional File 1. Study summary** Table

| **PART 1. INTERVENTIONS TO INCREASE YOUTH DEMAND FOR SRH SERVICES** | | | | | | |
| --- | --- | --- | --- | --- | --- | --- |
| **Study location and dates** | **Target population and objective** | **Evaluation** | **Description** | **Findings** | | **Effect size** |
|  |  |  |  | **Related outcomes:**  **Adolescents**   - **know when and why health services should be used;** - **know where health services can be obtained;** - **state intention to use services, if needed.** | **Primary outcome:**  **Use of health services** |  |
| ***In-school education*** | | |  |  |  |  |
| 1. Nigeria, Endo  (Okonofua et al., 2003)  Note: Peer education for adolescents took place in the community as well as in the school. | To improve uptake of ASRH services (for treatment of STIs) by in-school urban youths (high school 14–20 years). | Randomized controlled trial  -4 randomly selected intervention schools (and nearby STI treatment providers)  - 8 randomly selected control schools  -Pre (n=1896) and post (n=1858) intervention  surveys | - In-school health clubs - Specially trained health professionals provided   - IEC material on treatment and prevention of STIs);  - discussions, films.   - Peer education   - link to services (given list of those providing adolescent-friendly services).   - Linked health services   -providers trained (including pharmacists and private practitioners). | - Statistically significant improvements in knowledge of sexually transmitted diseases, including symptoms in intervention sites e.g. awareness if partner had an STD. - Statistically significant increased use of condoms in intervention sties. | - Statistically significant increase in use of STD services for males and females was found in intervention compared with control sites. This included increased use of private physicians for STI treatment (OR=2.1, 95% CI=1.1-4.0) and reduced treatment by pharmacists (OR=0.44, 95% CI=0.22-0.88). - Reported prevalence of STD symptoms in the past 6 months was significantly reduced in intervention compared to control schools (OR=0.68, 95% CI=0.48-0.95). | - Multivariate logistic regression with Huber's formula to account for school clusters. Odds Ratios and Confidence Intervals reported |
| 2. Brazil, Bahia  The Strengthening Public Sector Adolescent Reproductive Health Project  May 1997–Nov 1999  State Secretariats of Health and Education  (Magnani et al., 2001) | To promote responsible sexual and health-seeking behaviours, including use of public health clinics, among public secondary school students (focus on grades 6 & 8). | Quasi-experimental:  matched control group panel design  -6 pilot project secondary schools (4 in Salvador and 2 in interior of Bahia) paired with reference clinics and compared with matched (geographically, socio-economically and school size) control schools:  -KAP baseline and endline survey (n=1480) (high loss only 26% intervention tracked, 30% control – therefore analysed as independent samples).  - Service statistics from health facilities  -Clinic survey 1998 in 4 clinics (n=385)  -Teacher survey 1999 (n=34) | - School education   - inclusion of sex education in different school disciplines by trained teachers;  - student peer educators trained.  - cross-referral system between secondary schools and health clinics set up (contact between schools/teachers and health providers)   - Linked health services   -health providers trained (2 per clinic with one exception) | - No significant effect of project found on levels of sexual activity or contraceptive behaviour - Modest but significant increase in students citing health centre staff as potential sources of reproductive health and sexuality information. - Twice as many students knew about the referral clinics and, of those, 51% could correctly name the clinic, compared to 12% at the baseline. - Information about obtaining a family planning method was the key motivating factor cited by those going to clinics. - Marginally significant increase amongst girls in intention to use STI services in the future. | - 18% of teachers in intervention schools reported having referred at least one student to a reference clinic in the 1999 school year. - In the endline survey 2% of students at intervention schools reported having been referred to a clinic by a teacher and 10% reported having been to a referral clinic. This compares to the 24.3% who had attended any public health clinic in the previous six months. - Overall no significant increase in use of services amongst males OR=1.23 (95% CI: 0.16-9.7) or females OR=1.05 (95% CI: 0.19-5.76) related to the intervention. | - Multivariate logistic regression. Odds Ratios and Confidence Intervals reported. - Three potential sources of bias in estimates:  1. Schools and clinics chosen in part due to their willingness to participate 2. Before and after groups differ due to high drop-out and reasons for drop out were not collected 3. Logistic regression only controls for chosen variables and other factors may be correlated with the outcomes of interest |
| 3.Bangladesh Frontiers programme  1999–2003 (evaluation over two years, 2000–2002)  (Bhuiya et al., 2004) | To improve ASRH knowledge, attitudes and behaviour of in-school and out-of-school urban youths. | Quasi-experimental  Site A: Intervention  -Youth-friendly services  -Community interventions  Site B: Intervention (test additional effect of school education)  -Youth-friendly services  -Community interventions  -In -school education  Site C: Control.  -Baseline and endline population surveys (~6000 adolescents and 1500 parents) and qualitative interviews and focus groups.  -Service statistics | - School education   - led by teacher and peer supported  - trained to provide a participatory reproductive health curriculum tailored to in-school youth, and focusing on life skills.  Youth-friendly services  - providers trained.   - Community   - life skills education  - peer education  - sensitization and awareness-raising (sessions with gatekeepers, parents, teachers, leaders). | - Knowledge of acquisition of HIV, acquisition of sexually transmitted diseases, and pregnancy prevention improved in intervention and control sites (greatest increase in Site A, without the in-school intervention) - Adolescents in Site B (with school) were more likely to support use of contraceptives by unmarried adolescents than those in Site A - Adolescents in the site B revealed a more positive attitude towards health facilities for contraceptive and STI services compared with pharmacies - Use of condoms increased in intervention sites (greater improvement in Site B) | - Approximately one-fourth (4,729) of the adolescent population in the intervention catchment areas visited the adolescent friendly health facilities, including repeat visits. - Utilization of services doubled in site A and increased 10-fold in site B compared to the control. Use was 6 times greater in Site B compared with A. - Effect greater amongst in-school adolescents and lower for unmarried sexually active adolescents many of whom are not in school. | - Significance testing or multivariate logistic regression of changes in use of services was not carried out. - Evidence that increases were due to intervention is weak (i.e. differences in characteristics of intervention and control groups not controlled for and many other ASRH activities going on in the area). |
| 4. Senegal Frontiers programme  1999–2003 years (evaluation over 18 months)  (Diop et al., 2004) | To improve ASRH knowledge attitudes and behaviour of in-school and out-of-school youths aged 10–19 years. | Quasi-experimental  Tested additional effect of school education (as above).  -Baseline and endline population surveys and qualitative interviews. | - School education   - led by teacher and peer supported  - trained to provide a participatory reproductive health curriculum tailored to in-school youth, and focusing on life skills.   - Youth friendly services   - providers trained.   - Community   - peer education  - sensitization and awareness-raising (sessions with gatekeepers, parents, teachers, leaders). | - Proportion of adolescents knowing one or more contraceptive method rose significantly at intervention sites. Better knowledge of ways of using contraceptives, especially condoms, was noted at site B (with school) and knowledge of health facilities was significantly greater than the control. - Attitudes towards use of these methods improved, greater tolerance among unmarried adolescents. - Knowledge of health facilities increased at site B and at the control site, but not at site A where levels were already relatively high. - Intervention had no effect on use of contraceptives, including condoms (although there was some limiting of sexual activity). | - Visits to health facilities were low before the intervention. There was a significant rise across all three sites but levels remained modest – below 20% (e.g. amongst 15-19 yr olds: Site A boys 8%-13% (p<0.05) girls 12-14% (not sig); Site B boys 6-7% (not sig) girls 8-18% (p<0.05); Site C boys 9-12% (<0.05) girls 8-20% (p<0.05). Only increase at site B significantly (p<0.05) greater than the control and was more pronounced for older adolescents (15-19). | - Confidence intervals of changes in service use not reported - Multivariate logistic regression of use of services was not carried out. Evidence that increases were due to intervention is weak a large proportion of adolescents were found to be receiving ASRH information in the control, implying that intensive activities were also carried out in this area (by other groups) making it difficult to link findings directly to the intervention. |
| 5. Mexico ‘Frontier’  1999-3 years (evaluation over 18 months)  (Vernon & Dura 2004) | Improve ASRH knowledge attitudes and behaviour of in and out of school youths | Quasi-experimental  Tested additional effect of school education (as above)  -Baseline and endline population surveys and qualitative interviews. | - School education   - teacher led and peer supported  -trained to provide a participatory reproductive health curriculum tailored to in-school youth, and focussing on life skills   - YFS   -providers trained   - Community   -peer education  -sensitisation and awareness raising (sessions with gatekeepers, parents, teachers, leaders) | - Reproductive health knowledge, including of contraceptives and services, were quite positive to begin with and improved over the course of the intervention - No increase in utilisation of protection during sex - Positive trends that did occur were observed in both the intervention (no additional impact with school component) and control groups, suggesting improved attitudes, and behaviours were due to additional factors other than the project. | - No increase in use of health services. In the baseline and endline surveys adolescents were asked if they had visited a nurse or physician in the last 12 months and the proportion that had declined from 58% to 47%, with a similar decline observed in all three groups. At endline only 6.2% of those that had visited a provider had been for a reproductive health service. | - Significance testing or multivariate logistic regression of changes in use of services was not carried out. |
| 6. Kenya ‘Frontier’  1999-42 months (evaluation over 18 month implementation phase)  (Askew et al 2003) | Improve ASRH knowledge attitudes and behaviour of in and out of school youths | Quasi-experimental  (baseline and endline population surveys (~3700 (1000 boys) adolescents) and qualitative interviews  Test additional effect of school education  (as above) | - School education   -teacher led and peer supported  -trained to provide teach a participatory reproductive health curriculum tailored to in-school youth, and focussing on life skills   - YFS   -providers trained   - Community   -peer education  -sensitisation and awareness raising (sessions with gatekeepers, parents, teachers, leaders) | Contraceptive awareness significantly improved amongst adolescents who participated in either school or community interventions. However knowledge of some specific methods increased in all sites, so it is not clear the project had any additional effect.   - None of the interventions improved knowledge of how to use a condom. - Use of protection during sex remained low although modest increase amongst girls involved in school activities and in the control group. A decline in reports of pregnancy amongst unmarried adolescents suggests general social change affecting or enhancing the project’s results. | - No evidence of increased utilisation of services with few of the young people surveyed saying that they had attended a youth-friendly clinic (5%). | - Significance testing or multivariate logistic regression of changes in use of services was not carried out. |
| ***Community-based facilitated education sessions*** | | | | | | |
| 7.India, Pune, Maharashtra  (ICRW & KEM hospital research centre)  (Pande et al., 2007) | To provide reproductive sexual health education, care and counselling for married adolescents (14–25 years) and include a broad spectrum of community and family members. | Before and after study (no control)  - three components were all initiated simultaneously, and adolescents self-selected which to participate in;  - baseline survey (114 couples);  - process evaluation (qualitative);  - endline survey (74 couples). | - Education sessions in groups in the community led by trained volunteers - Professional counselling (one –on-one and couple) sessions in the community   Education and counselling aimed at young women, husbands, mothers-in law and others (community members informally participated in all activities). Education sessions included referral to counselling. Education and counselling included a referral system for those requiring clinical services.   - Youth-friendly services (provided by KEM)   -health providers trained | - Improved understanding of condom use as a way to prevent STIs and HIV. - Improved knowledge of need for antenatal care and recognition of danger signs in pregnancy. - High drop-out from education sessions but good uptake of   counselling. | - Increase in use of clinical services, e.g. for maternal health, infertility, family planning and reproductive tract infections (large percentage (70%) referred from health education sessions, 30% from counselling). | - Data not reported for increase in service use and no significance test carried out. No control. |
| 8.India, Maharashtra  (ICRW and Foundation for Research in Health Systems (FRHS)  2001–2006  (Pande et al., 2007) | Young newly-married couples (where wife is below 22 years) with a focus on the women. | Quasi-experimental  Intervention  - 1 site only social mobilization (demand)  - 1 only youth friendly services (supply)  - 1 both  1 control  Test supply versus demand approaches  - baseline and endline survey (1866 married girls)  - mid-intervention (972 husbands)  -75 mothers-in law, qualitative interviews)  - social mobilization (process evaluation)  -health worker/clinic records | - Social mobilization (SM) through existing community-based organizations (addressed low priority communities place on ASRH)   - strengthened youth and women’s groups (mothers-in-law and husbands drawn in to participate);  - adolescent and community education;  - community involvement in design and implementation.   - Youth-friendly services (addressed the fact that services are not geared towards ASRH)   - improve quality and accessibility of government services;  - sensitize providers to adolescent’s needs. | - All intervention sites showed similar increases in awareness of modern family planning methods and spacing. - Basic and detailed knowledge of maternal health, contraceptive side-effects and abortion increased most in the social mobilization sites. - The site with only youth-friendly services did not perform better than other sites on most outcomes. Although it was expected for social mobilization and youth-friendly services to be most successful, in many outcomes social mobilization alone performed better (possibly due to more focused efforts). | - Sites with social mobilization performed well in terms of increases in service use compared to those without. They performed best on post-natal checkups, contraceptive acceptance, treatment of gynaecological disorders, and treatment of STIs and reproductive tract infections. Treatment for STI/RTI increased 98.2% in SM/YFS site, 79.5% in SM only site, 44.5% in YFS only site and 26.7% in control. - The site with only GFS performed best only on care for high-risk deliveries. Treatment for high-risk deliveries increased 24.2% in SM/YFS site, 22.4% in SM only site, 44.5% in YFS only site and 26.7% in control. | - Data not reported for overall increase in service use and no significance test. Multivariate logistic regression not carried out. |
| ***Youth centres*** | | | | | | |
| 9. Rwanda, Butare  Centre Dushishoze  Population Services International  2001–ongoing  (Neukom et al., 2003) | Holistic approach to improve sexual behaviour and condom use, i.e. recognize that social support and self-efficacy are influential. | Before and after (cross-sectional surveys)  - Household Oct-Dec 2000 (n=3111)  -School survey Oct – Nov 2000 (n=1530)  - Household March 2002 (n=3109)  -School April 2002 (n=1555)  Examining trends in survey responses  after controlling for sample differences  and other confounding factors  such as education and socioeconomic  status and compare by level of exposure ‘dose response analysis’ | - Youth centre   - recreation and social activities  - vocational skills training  - information material  - peer education  - subsidized youth-friendly services (integrated RH and HIV)  - outreach days for parents and community members.   - Peer education   - education and counselling sessions in youth club, churches, schools, rural community centres  - identify and promote youth-friendly condom sellers in rural areas.   - Media campaign (social marketing)   - billboards  - newspaper  - mobile video unit. | - Increased confidence in condoms as an effective way to prevent HIV/AIDS. - Statistically significant increased knowledge of a nearby condom source and of where to find HIV testing and counselling services. - No effect on condom use | - Significantly higher utilization of HIV testing services with increased exposure for males and females. 7% of young women and 9% of young men ages 15 to 24 with high program exposure had an HIV test, compared with only 2% of those with low exposure (p≤.05) | - Change in service use related to exposure statistically significant at p≤.05 when age, residential area, level of education, school enrolment, socioeconomic status, and number of sexual partners controlled for. Confidence intervals not reported. - No control, analysis of effect of programme exposure suggests programme responsible for some but not all of the changes. |
| 10. Zimbabwe, Gweru  Pathfinder International  (Moyo et al., 2000) | To improve adolescent uptake of ASRH services | Before and after (no control)  -review of clinic data after a year. | - Youth centre   - recreation and services.   - Peer education. - Community sensitization   (initial stage)  - community meetings with leaders, parents and teachers.   - Youth-friendly services   - youth corners in clinics manned by peer educator. | - Improved attitude towards condom use. | - Use of youth centre but not for services. - No increase in use of youth-friendly services (although youth that visited were generally “satisfied” with the service). | - Service use data not reported in detail, no change exhibited so no significance test carried out |
| 11. Togo, Lome  (Kouwonou & Amegee, 2001; Speizer et al., 2004) | To provide a  supportive environment for youth; to  improve youth knowledge, attitudes, skills  and practices; and to increase service use  among youth (10–24 years). | Before/after (panel)  - baseline (2083 youth and 1027 adults) 1998  - follow-up 2000 (1679 youth) and 2001 (1332 youth plus 524 clinic users). | - ABTEF youth centre   - youth-friendly services (clinical and counselling)  - library  - education (literacy and vocational classes)  - recreation  - promotion in schools.   - Peer education   - outreach education and referral.   - Media   - radio and television promotion (discussions and round table discussions). | - Some increase in knowledge of condoms. - Media exposure was positively associated with use of the youth centre. - Youth who visited the youth centre over the follow-up period were significantly more likely to be condom users than youth who either never visited the centre or had already visited the centre at observation 1. This suggests that, for contraceptive users, visiting the centre affirms contraceptive use behaviours. | - Moderate (non-significant) increase in use of youth centre, including services, over time (baseline 3.3% reported ever visiting the youth centre (for recreation or clinical services) and by 2001, 10.3% had). - Youth exposed to a peer educator (Logistic Regression Co-efficient (LRC)= -1.31 p<0.001) or who lived nearer (LRC=-2.52 p<0.001) or were exposed to media (LRC=-0.72 p<0.01) were significantly more likely to have visited the youth centre. - Young people in the target population were more likely to visit the youth centre clinic than the other clinics in the area (LRC=1.53 p<0.001). | - Use of youth centre for services versus recreation not investigated separately over time - Some problems   encountered obtaining follow-up data i.e. biased compared to representative baseline sample. Follow-up sample more likely to be male, in-school and more educated, all factors possibly associated with the outcomes of  interest (use of the youth centre and contraceptive use).   - No control. |
| ***Information, Education, Communication (IEC) outreach from health facilities*** | | | | | | |
| 12. Thailand  The PATH *RX Gen* programme  PATH Thailand  (Bond, Firestone and Francis, 2003) | To improve the quality of ASRH services through training and consumer education. | Before and after  - clinic data | - Youth-friendly services   - pharmacists and drug sellers trained to improve service quality for youth (meetings and training sessions);  - guidelines for youth-friendly services developed (provide information about products, history-taking and referral, and guidance to improve interpersonal communication with young clients);  - RX Gen logo shows adhering to guidelines;  - establishment of a referral network to social and health services (public counselling, health centres, social services, vocational training centres).   - IEC   - information booklets and referral cards distributed at drugstores and in the community at fairs and shopping centres;  - information about webpage with links to the Ministry of Health’s service network;  - IEC and promotion by radio. |  | - After the referral cards were disseminated widely and promotion began, the number of young clients utilizing the services at participating drugstores increased, with an increase in the number of clients who sought reproductive health advice from the pharmacists. - Increase in referrals by pharmacists to counselling services - Data from government health centres (part of the referral network) indicated a two-fold increase in the number of young clients seeking related services following the establishment of the referral network. | - Detailed service data not reported and no significance test - No control |
| 13. Madagascar, Tamatave Province  TOP Reseau (“top quality cool network”) Youth-friendly social franchise project  Population Services International  2000–ongoing  (LaVake, 2003; Neukom & Ashford, 2003) | Community-wide intervention aimed at preventing HIV/AIDS and unplanned pregnancies  by motivating sexually active youth (15–24-year-olds) to treat STIs and to use condoms  consistently or not have sex. | - Before/after (no control)   - household surveys  - clinic data. | - Youth-friendly services   - private provider social franchise model (clinics, pharmacies and others);  - quality standards;  - provider training;  - subsidies;  - marketing and promotion of services (17 clinics). E.g posters, radio spots and mobile video units–   - Peer education (paid full-time educators)   - reach diverse locations via mobile units;  - small group and individual counselling sessions;  - group discussions;  - health films.   - Mass media   - social marketing, radio and television.   - Community education sessions   - parents, religious leaders, teachers, school administrators. |  | - Clinic attendance records indicated increased utilization in the first two years – 527 first quarter of 2001 to 2202 fourth quarter of 2002 (these were predominantly females (89%), and a subsequent focus has been increasing male uptake of services) and about half of visits were for reproductive health services. No age breakdown is currently available but more detailed tracking is beginning (it is thought that ~60–70% are youth). - Success has led to funding from the Global Fund to expand to three new provinces. | - No significance test of increase in service use - No control |
| 14. Mexico  Mexfam, *Gente Joven*  1994–ongoing  (LaVake, 2003) | Adolescents in communities with high levels of poverty | - Before/after   -surveys  - clinic data. | - Joint venture social franchise model. Mexfam joined with private providers and other NGOs as franchisees. - Youth-friendly clinics   - provider training;  - subsidized services;  - franchise agreement;  - logo/branding.   - Youth centres   - recreational activities;  - some have clinical services   - Peer education   - young people trained as promoters, counsellors, contraceptive distributors  providing school and community outreach.   - Community   - youth engage support from the community;  - youth involved in programme development and management committees.   - - Mexfam staff provide school outreach. | - Increased contraceptive use at last sex. | - Youth services that are   integrated into adult programmes, both at Mexfam centres and private clinics, seem to be reaching more young people than clinics at Mexfam youth centres. | - Detailed service data not reported and no significance test - No control |
| 15. Mongolia  3-year project (1999-2003)  (WHO, 2003) | To increase adolescents’ (10–19 years) access to quality health services | Quasi-experimental  (2 rural districts  and 3 districts in capital)  - assessment after 1 year of application of YFS criteria and in-school youth survey  - service statistics Jan-June 2003 | - Youth-friendly services   - quality standards;  - train staff;  - improve equipment/supplies (including contraceptives);  - improve confidentiality.   - IEC   - IEC materials developed and distributed.   - Community advocacy and mobilization   - governors, teachers, health workers, parents and adolescents targeted;  - adolescents involved in design of education and advocacy material. |  | - Statistically significant increase in use of services 10-19 year old males (OR= 1.3 p<0.05) and females (OR=1.8 p<0.05) in project sites significantly more likely to visit clinic than in control sites. - Evaluation does not allow the impact of IEC activities and wider community mobilization to be gauged separately. | - Significance test carried out but not multivariate logistic regression -results were not controlled for differences in catchment populations. |
| 16. China, Songijiang district, Shanghai  (Chao-Hua et al 2004) | To build awareness and to offer counselling and services related to sexuality and reproduction of unmarried urban young people aged 15–24 years both in and out of school | Quasi-experimental  (baseline survey n=1220 intervention n=1007 control, follow-up survey 20 months later n=1148 intervention n=894 control)  1 intervention and 1 control community. | - Youth-friendly services   - youth health counselling centre (with contraceptive services);  - service providers trained;  - free condom supplies.   - IEC   - information activities and materials about SRH and availability of services made available in the community;  - sex education lectures;  - interactive discussions;  - videos shown before cinema films.   - Community sensitization   - meetings for community leaders and parents. | - Increase in knowledge of available services (data not reported). - Increase in contraception and condom use at interventions site (Logistic regression – after adjusting for demographic factors intervention group were more likely (OR=14.58 95% CI 8.55-24.87 p<0.001) to use contraceptives than those from control groups). |  | - Data for increase in knowledge of available services not reported and no significance test carried out. |
| ***Life skills education or broader youth development approaches*** | | | | | | |
| 17. India  Better Life options Programme (BLP)  Bhartiya Grameen Mahrasangh with support from CEDPA  1996–1999  (Levitt-Dayal & Motihar, 2000) | Youth development approach (holistic empowerment model) to tackling the numerous concerns and needs (health, economic, education) in married and unmarried adolescents (12–20 years) | Quasi-experimental post-test only, after 3 years of intervention. Structured survey n=1693 girls (858 controls and 835 BLP alumnae) | - Life and livelihood education   - non-formal, participatory education in village training centres run by local literate women.  • Holistic empowerment model focusing on youth development  - reproductive and sexual health;  - vocational skills;  - literacy;  - basic skills for living (nutrition, self-protection from violence);  - family life skills;  - leadership skills;  - personality development;  - challenging gender inequalities;  - recreation.   - Community   - community involvement in development of programme. | - Improved knowledge about reproduction and contraceptives. - Increased literacy, employment, communication and autonomy (e.g. about spending what they earned). - More likely to discuss family planning with their husbands and to use contraceptives (and greater intention to use them in the future). | - Compared with those in the control group (21%), significantly (p<0.001) more BLP girls (68%) had travelled outside their village and went to a health centre alone in the last six months. - Compared to controls, the BLP alumnae were more likely to have used prenatal, delivery and postnatal care in their last pregnancy. | - Confidence intervals for change in service use not reported and no multivariate logistic regression carried out - Post-test only |
| 18. India, Delhi “Swaasthya” programme  ICRW  April 1998–April 2001 (Tigri pilot)  July 2003–July 2006  (Naglamachi slum- replicability study)  (ICRW, 2005, 2007; Pande, 2007) | To enhance the sexual health and decision-making of adolescent girls (targeting unmarried girls aged 12–22 in Tigri (an area with many economic migrants from surrounding states) and both married and unmarried girls in the Naglamachi slum | Before/after surveys (no control)  Tigri (n=401) and Naglamachi (n=295 baseline, 365 endline) and qualitative interviews with community members and Swaasthya staff members  - analysed by intervention component exposure  Sustainability study in December 2005 (to see if changes were sustained after Swaasthya withdrew in April 2001) , Tigri | - Life skills education, participatory approach. Holistic youth development model   - seven skills-building modules;  - reproductive and sexual health was one module.   - Development of a social and peer support network (including supportive adults)   - women’s groups for adolescent girls and their mothers to increase understanding between them;  - involved community elders and boys as well as mothers.   - IEC dissemination   - one-on-one interaction with Swaasthya female health worker;  - television programmes and videos on community and adolescent issues (Tigri only, as not found to be effective). | - Improved knowledge of reproductive and sexual health including contraceptive use and availability and use of services. Tigri: high knowledge of SRH issues 57.7% in participants 53.9% in non-participants. Naglamachi: 62.2% in participants, 43.3% in non-participants - However, overall effects were weaker in Naglamachi than in Tigri. The former has a more conservative environment with girls having less freedom to attend programmes. Earlier work in Tigri by Swaasthya may also have made people more receptive. - The sustainability study showed that the community did not sustain all the components of Swaasthya, and that knowledge of sexual and reproductive health decreased, suggesting that consistent input is needed to maintain knowledge. |  | - Use of services not measured, no significance test of change in knowledge of services - No control |
| ***Use of Media*** | | | | | | |
| 19. Zimbabwe  (Kim et al., 1998, 2001) | To promote sexual responsibility among youth aged 10–24 years living in cities or in the centres of small towns in rural areas | Quasi-experimental (before/after n=1400)  5 intervention and 2 control areas | - Media   - radio;  - launch events;  - drama events;  - hotline;  - promotion of services.   - Peer education   - including referral.   - Information material distributed at schools. - Youth-friendly services   - trained family planning providers. | - Improved knowledge and discussion of sex - Contraceptive use at last sex rose significantly in campaign areas (from 56% to 67%). | - Service use increased in campaign areas. Logistic regression (controlling for respondent’s age, sex, education, sexual experience, marital status and urban-rural residence) showed young people in campaign areas were 4.7 (OR=4.7 p<0.0001) times more likely to visit a health centre. | - Multivariate logistic regression of impact on service use but Confidence Intervals not reported and contamination of control weakens evidence. |
| 20. Burkina Faso  Advocates for Youth and Pacific Institute for Women’s Health  4-year programme 1998–2002  (Yaro et al., 2003 & 2007) | Community mobilization to help identify and tackle local priorities (includes use of services and communication regarding ASRH) | Before/after (no control)  - survey | - Media   - folk and modern.   - Peer education   - discussions;  - home visits;  - role plays.   - Community   - awareness projects for parents and other community members;  - community involvement in developing, implementing and evaluating programme. Local organizations serving young people worked with community members in 20 villages to develop action plans based on local needs.   - Youth-friendly services   - providers trained;  - adjusted operating hours;  - special youth area. | - Knowing how to use a condom correctly (up from 52% to 84%). - Aware of where to obtain health services (up from 62% to 78%). - Increased proportion of sexually active youth reporting current condom use (up from 51% to 73%). |  | - No significance testing - No control |
| ***Voucher finance interventions*** | | | | | | |
| 21. Nicaragua, Managua and the departments of Rivas and Chinandega  Instituto Centro Americano de la Salud (ICAS) and the London School of Hygiene and Tropical Medicine  (Meuwissen et al., 2006a & 2006b) | Aimed at all poor female adolescents aged 12–20 years of age to increase their utilization of sexual and reproductive health services and overcome obstacles to seeking care | - Quasi-experimental   - community-based survey of voucher recipients (904) and control (2105)) | - Youth-friendly services   - 28 711 vouchers distributed in disadvantaged neighbourhoods, at markets and in schools directly by ICAS and through a network of youth NGOs;  - the vouchers offered free access to sexual and reproductive health services in 20 health centres in Managua.  • Provision contracted to public, NGO and private providers. Services included counselling, family planning, pregnancy tests, prenatal control, and diagnosis and treatment of STIs according to the needs of the adolescent | - Improved knowledge of contraception and STIs and their prevention in voucher recipients. - Increased condom use at last sex among recipients (especially those surveyed outside schools). - Increased contraceptive use among in-school voucher recipients. | - Half of the 1025 sexually-active girls who received a voucher used it, compared with only 14% of girls who were not sexually active. - Voucher receivers (34%) had a significantly higher use of services than nonreceivers (19%) (adjusted odds ratio – controlling for differences in study populations= 3.1 95% CI 2.5-3.8) - Highest influence in school where use was 24% relative to non receivers (adjusted odds ratio 5.9 95% CI 3.7-9.5). Girls who were younger, or less well educated also benefitted more. | - No randomisation but multivariate logistic regression carried out. |
| 22. Kenya  “Friends of Youth” Health Project  Family Planning Association of Kenya and the Population Council  1997–2001  (Erulkar et al., 2004) | Community-wide intervention aimed at improving ASRH knowledge, attitudes and behaviour in urban and rural youth aged 10–24 years in and out of school | Quasi-experimental  (Baseline survey 1997, endline survey 2001) | - Media. - Peer education (“friends of youth”). - Community   sensitization and involvement.   - Youth-friendly services   - service provision was contracted out to private and public service providers, skills of the providers were updated, and staff received training in “youth friendliness”;  - no written contract was entered into with providers.   - Finance   - use of vouchers (2800 distributed) for subsidized youth friendly services;  -peer educators distributed vouchers directly to adolescents in need. |  | - Most of the 2772 vouchers used were for STI services (55%), followed by family planning (15%) and male circumcision (15%). - Voucher recipient vouchers were followed up and encouraged to use meaning high rate of service use compared with control areas | - No comparison of service use made between intervention area and control – high uptake in intervention area due to follow up of voucher recipients |
| ***Multi-component and Multi-sectoral approaches*** | | | | | | |
| 23. Jamaica, Youth Now Adolescent Reproductive Health Program  (in collaboration with YMCA and others)  2000–ongoing  USAID-funded  (Tiffany et al., 2003 mid-term evaluation; Russell-Brown, 2003) | To implement ASRH policies and create a supportive social environment to improve ASRH outcomes.  To test a variety of approaches to ASRH information and service provision (traditional health centres shunned due to lack of privacy). | Before/after (national, no control)  -Surveys  2000- baseline  2001 and 2002 follow up  - Clinic data  -Process evaluation  -Qualitative data | - School and community education and counselling   - peer educators, school nurses, girl guide leaders and coaches provide education, counselling, condom distribution and referral.   - Media campaign   - included promotion of a helpline.   - Parenting education. - Intensive work with the church   - workshops, consultation and training.   - Youth-friendly services (NGO and public)   - providers trained  - health centres linked to schools.   - Advocacy and policy development   - parish and national level. | - Improved attitude of young people to services available. | - Service utilization increased (small decrease after 1 year but increased thereafter). Year 1 compared with baseline 2.6% reduction in adolescents using services, Year 2 compared with Year 1: 123% increase in adolescents using services and 59% increase in use of family planning services - Importance of information and communication acknowledged. | - No significance tests detailed data not available |
| 24. Mozambique  Geração Biz, government programme  UNFPA, Pathfinder  Started in 2 districts (Maputo and Zambezia), expanded to 6, and aims for national coverage  1999–ongoing  (Senderowitz et al., 1997; Hainsworth 2002) | In-school and out–of-school 15–24-year-old youths | Before/after  KAP surveys – knowledge, attitudes and practice and clinic data  Community mapping exercises | - School   - peer education (films, drama, group debates);  - teachers trained, national curriculum being developed;  - links to youth-friendly services;  - life skills education.   - Community   - outreach education, including  peer education (films, drama, group debates);  - advocacy work to explain need for ASRH services to community members;  - support for HIV-positive youth.   - Media   - community radio programmes.   - Youth centres   - receive counselling, condoms, referral to services.   - Youth-friendly services   - providers trained;  - peer education.   - Youth involvement in designing and implementing programme. | - Condom use increased by an average of 28% in the two initial districts. | - Number of adolescents aged 15–19 visiting clinics for counselling and services increased more than 10-fold in Maputo (1173 at baseline in 1999, 11,726 in 2000). Number of young men served almost doubled from 10% to 19% in 2000 - In Zambezia service statistics not available for 1999, but in 2000 number rose to 11,669. A larger proportion of clients were male (39%) than in Maputo. - Counselling and contraception reported as the two most popular services in both areas. - Adolescents were concentrated in the older age groups and more females than males visited. School students were also well represented, demonstrating the impact of school links with youth-friendly services. There was a delay in implementation of outreach activities, which resulted in weaker community links. | - No significance testing - No control |
| 25. Ethiopia Addis Ababa and Oromia regions  Initiative to Save Young Generation’s Health Today (INSYGHT)  Save the Children, October 2003 to September 2006 (school programme been under way in Addis Ababa since 1997)  USAID-sponsored  (Gebregiorgis et al., 2005) | In and out of school youths including primary school age (under 11) | Before/after evaluation  Process evaluation of ASRH clubs (surveys and interviews)  Evaluation of utilization of youth-friendly services (document review, key informant interviews, clinic records) | - In-school and out-of-school ASRH clubs (primary and secondary)   - peer education;  - songs, literature, assemblies, audio/visual media;  - home visits to married youth by out of school club members;  -youth action kit (IEC materials).   - Community   - outreach community activities and events by peer educators to reach community and religious leaders, government representatives, elders, shopkeepers and parents (dialogue with stakeholders for involvement in programme)  - parent training, with active interested parents trained as peer educators;  - orientation sessions with school principals;  - community events;  - radio programmes.   - Youth-friendly services   - increase availability, accessibility and quality of health services. | - Improved knowledge of family planning. - Improved knowledge of sources of family planning, counselling, and STI/HIV services and what to do in case a medical consultation is needed. | - Service statistics show uptake of family planning and reproductive health services including voluntary counselling increased. | - No significance test - No control – other ASRH activities taking place in the area |
| 26. Bhutan, Malawi, Nepal, and Vietnam Save the Children, USA  (Save the Children, 2005)  1999–2004 (ongoing activities in various forms) | To improve access to quality services, empower adolescents (10–25 years) to make positive life decisions. To build social and political capital for ASRH with a focus on changing community norms. | Before/after  Quantitative and qualitative evaluation (group discussions and semi-structured interviews) | - Participatory approach   - youth and community involvement in planning, implementing and evaluating activities.   - Peer education and youth action teams. - Youth clubs/information centres   - youth forums;  - peer education;  - IEC and counselling.   - Life skills education   - integrating into scout programme.   - Media   - newsletter;  - magazine.   - Youth-friendly< services   - availability and quality;  - dialogue between youth and health service providers;  - clinic and outreach.   - Community mobilization   - parents, religious leaders education;  - community gatherings;  - street theatre.   - Policy advocacy   - work with governments. | - Improved knowledge of contraceptives and condoms for preventing HIV and pregnancy. Increased knowledge of available services. - In Malawi statistically significant differences (p< .05) in sexual behavior among young men and women exposed to the program emerged. Approximately 22% of young women in the exposure group mentioned having changed their behavior since hearing about HIV/AIDS by avoiding multiple partners, compared to 15% of   those in the non-exposure group. 21% of exposed males mentioned that they had begun to use condoms, compared to 12% of non-exposed males.   - In Nepal need for more youth-friendly service centres identified by adolescents . | - Increased uptake of condoms and ASRH services. E.g. In Malawi improvements in services and an increased awareness of risks and treatment contributed to an increase in the number of female STI clients between April 2002 and April 2004. | - Detailed service uptake data not reported and no significance testing carried out - No control |
| 27. African Youth Alliance, partnership between UNFPA, PATH and Pathfinder  Ghana, Tanzania, Uganda and Botswana  (financial constraints meant the programme finished 2 years early)  2000-2006  (resources are being mobilized for a second phase) | Seeks to improve sexual and reproductive health of young people, aged 10–24 (with emphasis on 10–19), in Botswana, Ghana, Tanzania and Uganda | Baseline surveys (2001–2002), mid-term assessments (2003), component evaluations (2005–2006) and independent impact survey evaluation (2006–2007) | - Policy and advocacy   - develop enabling environment for ASRH.   - Behaviour change communication   - first stage highly participatory to increase awareness of ASRH in communities (e.g. work with religious groups);  - extracurricular ASRH education in schools;  - peer education;  - mass media  e.g. in Ghana the "Challenge Cup" used the appeal of football to promote youth-friendly sexual and reproductive health information and services,   - Livelihood programme   - life planning skills, education and counselling, both in and out of schools (including strategies to reach vulnerable subgroups).   - Youth-friendly services   - partnering with facilities (static and outreach) to improve quality;  - work with NGOs, faith-based organizations, public facilities and non-traditional providers (including peer service providers);  - including outreach and promotion.   - Institutional capacity-building. - Coordination and dissemination. | - Increase in condom use by females (first sex, last sex, ever use, always use) in all countries. - Significant increase in modern contraceptive use by females (first sex, last sex) in all countries Condom use (first sex, always use, ever use) and modern contraceptive use (first sex) increased among males in Tanzania (no impact in Ghana). | - Youth-friendly services clinic and outreach utilization increased. E.g. in Tanzania emerging trends show more girls are accessing services. E.g. in Uganda approximately half of the clients seen at AYA-supported clinics are new clients and two thirds of all clients are out-of-school. - Heterogeneity of young people is also evident in their preferences for service provision. Data   across all countries showed that more females visited clinics for counseling, more males obtained condoms through outreach. Variation also exists among age groups. Increasing  utilization of services requires multiple approaches to match diversity of youth. Outreach work is essential to create demand for clinical sexual and reproductive health services, especially among men (reduce stigma).   - In general, a large increase in demand for ASRH education, information and services has begun in all countries, fuelled by AYA’s multiple strategies of behaviour change and communication. | - Data on service use not reported in detail or analysed, focus placed on contraceptive use and changes in knowledge |
| 28. RHIYA  Funded by European Union (EU),  UNFPA-led, with many local partners  2003–2007  (following on from the EU-UNFPA Reproduction Health Initiative 1998–2002)  Being implemented in seven Asian countries (Bangladesh, Cambodia, Laos, Nepal, Pakistan, Sri Lanka and Vietnam).  (EU/UNFPA, 2006, 2007) | To improve  the sexual and reproductive health (behaviour, practices and awareness) of young  people, aged 10–24, including utilization of services | Before/after (no control)  Clinic and youth centre attendance data | - Advocacy   - increase political and  community support for adolescent sexual and reproductive health.   - Behaviour change communication   - partnering with young people and communities to increase awareness and to improve sexual and reproductive health among adolescents and youth.   - In-school education   - e.g. teachers trained   - Peer education   - e.g. in school, in community, including life skills workshops  - e.g. bar workers trained in adolescent reproductive health and life skills   - IEC materials   - e.g. distributed in village development committees   - Youth clubs   e.g. become youth information centres and provide peer education, counselling, clinical services and vocational training   - Counselling   - e.g. telephone counselling   - Community sensitization   - e.g. sensitization workshops and support groups formed  community health, communication sessions. Parent, teacher and community leader groups set up with regular meetings, plus meetings of stakeholder group  - e.g. street theatre   - Youth-friendly services   Working to improve access to quality  youth-friendly sexual and reproductive health services  - e.g. “condom cafes” at work  - e.g. yfs at government health centres , in some cases separate male and female centres   - Institutional capacity-building   - developing the technical, planning and managerial capacity of government and local civil society organizations to meet the sexual and reproductive health needs of young people. | - Significant improvements in awareness and knowledge of STIs across all RHIYA countries. This was identified as a particular problem at   the start of the programme.   - Improvements in knowledge of condom use. - An overall narrowing of gender gaps in sexual and reproductive health knowledge and behaviour across RHIYA countries. - Strong and significant improvement in contraceptive use, particularly condom use (e.g. Vietnam where 24% used condom at last sex before intervention, and 48% after). | - Demand for and utilization of health services reported to have increased in many countries.   Examples:  In Bangladesh utilization of health services increased since the beginning of the project but challenges remain to make the service accessible to all;    In Nepal utilization of services in health facilities and youth information centres has increased;  In Vietnam it has been acknowledged that youth corners could benefit from increased publicity (being addressed in Phase II), plus plan to make youth-friendly services available on a broader scale as part of public health facilities.  In Pakistan youth attendance was low at first. Further outreach and sensitization was carried out and the centres’ operating hours were adjusted, and young men gradually started attending the male centres. Girls, however, still faced restrictions on their mobility. To address this, further door-to-door outreach was done to contact parents and motivate them to allow their daughters to participate in RHIYA activities. | - Detailed service uptake data not reported and no significance testing carried out - No control |
| 29. Nepal  12–24 months of intervention (November 2000 to March 2003)  (Mathur et al., 2001) | To improve ASRH health of urban and rural young people, and test influence of youth and community participation in programming | Quasi-experimental  - baseline and endline surveys (households, adolescents and service providers);  - mystery client survey;  - qualitative data collected at baseline and endline. | Intervention site (greater youth and community participation throughout design and implementation)   - Youth clubs. - Livelihood training. - Community education   - adult education;  - street theatre.  Control site   - Peer education - Teachers trained to provide education. - Youth-friendly services. | - Improved articulation of specific reproductive concerns (e.g. side-effects of contraception, symptoms of reproductive tract infections and STIs). - The understanding of service options available and how to use them was greater in the intervention areas. - Clear male/female differences. Strong evidence for gains in young women’s knowledge, and reduction in sexual activity among rural young men. A moderate negative effect on knowledge of rural young men and sexual behaviour of urban young men. - Higher demand for reproductive health information and services (adults and youth) and in-depth understanding of issues in the intervention areas. | - Delivery in medical facility increased at both sites but was much more substantial at the study site (Control – increase from 11.8 to 22.5%, Study – increase from 17.4 to 45.0%) - The proportion of young women seeking prenatal care for a first pregnancy increased substantially at the study site but in contrast the control showed a slight decline (Control – decrease from 41.2 to 36.6%, Study – increase from 4.8 to 66.7%) | - No significanct test conducted - No multivariate logistic regression |
| **PART 2. INTERVENTIONS TO INCREASE COMMUNITY SUPPORT FOR SRH SERVICE USE BY YOUNG PEOPLE** | | | | | | |
| **Study location and dates** | **Target population and objective** | **Evaluation** | **Description/Setting** | **Findings**   - **Awareness of adolescents' need for health services.** - **Approval of service provision and use.** - **Action taken to improve service provision to adolescents.** - **Action taken to improve service use by adolescents.** | | **Effect size** |
| ***Community sensitization via multimedia*** | | | | | | |
| 1. Zimbabwe  (Kim et al., 1998, 2001) | To promote sexual responsibility among young people aged 10–24 years living in cities or centres of small towns in rural areas | Quasi-experimental (before/after survey n=1400)  5 intervention and 2 control areas | - Multimedia   - radio;  - drama events;  - hotline;  - promotion of services (information material distributed in schools).   - Peer education   - including referral.   - Youth-friendly services   - family planning providers trained. | - - Traditionally, aunts, uncles and other members of the extended family provided sexuality-related information to young people. However, as urbanization increases the distance between family members, parents are taking greater responsibility in this area. Many parents feel uncomfortable in this role. As a result of the campaign, 80% of respondents had discussions about reproductive health, with friends (72%), with siblings (49%), with parents (44%), with teachers (34%) or with partners (28%).   - Concluded that a multimedia approach is an effective way to build community support for behaviour change. It helps to ensure that young people find approval for their actions and have access to services. Decentralizing management to local committees that included representatives from local government, religious, educational, health and business groups; designing activities to reach a secondary audience of family, friends and teachers, and to prompt discussion of reproductive health issues; and by involving providers in campaign preparations and launches all contributed. | | - Logistic regression analysis - Limitation: contamination of control weakens evidence. |
| ***Community participation and mobilization*** | | | | | | |
| 2. Zambia, Lusaka  Lusaka District Health Management Team, with Care, UNICEF and John Snow International  1994–ongoing  (Nelson & Magnani, 2000; Mmari & Magnani, 2003) |  | Quasi-experimental  -8 intervention sites and 2 control sites  - qualitative interviews (peer educators, service providers, young people etc);  - service statistics.  All clinics in intervention sites improved their youth-friendliness and to varying degrees involved the community in activities (degree of friendliness and level of acceptance of provision of ASRH services measured). | Varying degrees of community participation   - Community mobilization (sensitization)   - “participatory learning for action” activities carried out to varying levels (e.g. use of role plays, debates and small group work).   - Peer education   - chosen either by the programme, by young people or by neighbourhood health committees.   - Youth-friendly services   - trained providers  - improved privacy, confidentiality etc. | - Mobilization activities led to fewer negative beliefs (e.g. belief that family planning services are for married adults). - Positive correlation between community acceptance of the provision of ASRH services (Spearman’s Rank Order Correlation: family planning (0.43), outpatient visits (0.41), and reproductive health services (0.35)) and their use, but not signicant at 95% level (low statistical power – 22%). In contrast only one of the correlations between youth-friendliness and service use was positive (family planning 0.32). - While some barriers to service use can be tackled through supply side interventions, the family, social, peer and community influences on care-seeking are very important. | | - Significance testing carried out but statistical power limited because of the small number of clinics and respondents, meaning relatively high risk of failing to detect significant relationships when they existed. - Not possible to control for unobserved differences amongst clinics that may have predisposed some to attract higher levels of clients or unobserved community level factors that may have differentially predisposed youth to using clinic services in some communities. - Non probability sampling methods were used to select the clinics |
| 3. Jamaica,  Youth Now Adolescent Reproductive Health Programme  (in collaboration with YMCA and others)  2000–ongoing  USAID-funded  (Tiffany et al., 2003, mid-term evaluation) | To implement ASRH policies, to create a supportive social environment to improve ASRH outcomes, and to test a variety of approaches to ASRH information and service provision (traditional health centres shunned due to lack of privacy). | Before/after (national, no control)  -Surveys  2000- baseline  2001 and 2002 follow up  - Clinic data (national, no control)  -Process evaluation  -Qualitative data | - School and community education, counselling and mobilization   - peer educators, school nurses, girl guide leaders and other coaches provided education, counselling, condom distribution and referral;  - intensive work with the church (workshops, consultations and training).   - Media campaign   - included promotion of a helpline.   - Parenting education. - Youth-friendly services (NGOs and public)   - providers trained.  - health centres linked to schools.   - Advocacy and policy development   - parish and national level. | - Achieved good community awareness of the programme - Partnership with all community influencers considered essential to gain acceptance and to change community values, attitudes and norms in support of adolescent reproductive health. - Improvements in the traditionally negative attitudes towards adolescent sexual activity that affects access to information and services (e.g. pastors’ and church leaders’ awareness was raised concerning the tension between theological theory and realities of adolescents lives, leading them in turn to train other church members; church leaders promoted adolescent services and encouraged their use). | | - Qualitative data supporting changes in community acceptance – quantitative survey data not available for indicators of community acceptance |
| 4. Geração Biz, Mozambique, government programme  UNFPA, Pathfinder  Started in 2 districts, expanded to 6, aiming for national coverage  1999–ongoing  (Senderowitz et al., 1997; UNFPA and Pathfinder International) | In-school and out-of-school 15–24-year-olds | Before/after  KAP surveys – knowledge, attitudes and practice and clinic data  Community mapping exercises | Youth involvement in designing and implementing programme   - Community   - outreach education, including  peer education (films, drama, group debates);  - advocacy work to explain the need for ASRH services to community members;  - parents trained as community activists.  - support for HIV-positive youth.   - - School   - peer education (films, drama, group debates);  - teachers trained (national curriculum being developed);  - links to youth-friendly services;  - life skills education.   - Media   - community radio programmes.   - Youth centres   - provide counselling, condoms, referral to services   - Youth-friendly services   - providers trained;  - peer education;  - dialogue between young people and health service providers. | - Increased understanding of need for ASRH services among community members through outreach education and sensitization. - Improved parent-child communication - Community support for implementation of the programme was established. Involving parents as community activists helped to create a supportive environment for the provision of sexual and reproductive health services and information, and helped facilitate communication between parents and their children. | | - Anecdotal and qualitative evidence |
| 5. African Youth Alliance, partnership between UNFPA, PATH and Pathfinder  Ghana, Tanzania, Uganda and Botswana (financial constraints meant the programme finished 2 years early)  2000-2006  (resources are being mobilized for a second phase) | To improve sexual and reproductive health of young people, aged 10–24 years (with emphasis on 10–19 years), in Botswana, Ghana, Tanzania and Uganda | Baseline surveys (2001–2002), mid-term assessments (2003), component evaluations (2005–2006), and independent impact survey evaluation (2006–2007) | - - Behaviour change communication   - drama, debates, festivals, sports events, peer education, extracurricular ASRH education in schools, mass media, youth clubs and parent–child communication sessions;  - first stage highly participatory to increase awareness of ASRH in communities (e.g. work with religious groups).     - Livelihood programme   - life planning skills, education and counselling;  - in school and out of school (including strategies to reach vulnerable subgroups).   - Policy and advocacy   - develop an enabling environment for ASRH.   - Youth-friendly services   - partnering with facilities (static and outreach) to improve quality.   - Institutional capacity-building. - Coordination and dissemination.   Cross-cutting objectives:partnerships, youth participation, gender, community involvement, sustainability and scaling-up. | - - Improved knowledge and supportive attitudes of young people and stakeholders towards ASRH intervention, including services. - Behaviour change communication   Tanzania: The media partnership programme reached millions and resulted in increased awareness of ASRH, increased leadership support for ASRH issues, more openness in public discussions on sexuality and condoms, and increased youth participation in activities.  Ghana and Tanzania: Football has provided an entry point for promotion of ASRH issues and for involving the community.  Botswana: Interactive local dramas, dance troupes (led and managed by young people), radio shows and mass media campaigns contributed to fostering of community awareness and positive community change (e.g. support and praise from leaders, teachers and parents). They also helped young people to feel more comfortable to talk about ASRH issues with their parents, teachers and service providers.  Non-traditional condom distributors (e.g. young barbers, artisans and shopkeepers)  Ghana: These distributors contributed to creating more positive views in the community toward ASRH information and service provision   - - Increased commitments and actions supportive of ASRH and use of services by stakeholders   Uganda: The Mufti of Uganda announced that Muslim couples should use condoms in marriage to prevent HIV/AIDS and other STIs. The Anglican Church signed a declaration supporting ASRH and revised prenuptial counselling guidelines to include voluntary counselling and testing  Botswana:Religious leaders supported discussion of ASRH issues and provision of services, and encouraged community support for them. Support from leaders, teachers and parents helped make young people more comfortable when talking about ASRH issues with them and with service providers.   - Challenges of tackling the sensitivity of ASRH, fostering ownership and reaching diverse segments of the population acknowledged | | - Qualitative evidence |
| 6. RHIYA  Funded by EU,  UNFPA-led, with many local partners  2003–2007  (following on from the EU-UNFPA Reproduction Health Initiative 1998–2002)  Being implemented in seven Asian countries (Bangladesh, Cambodia, Laos, Nepal, Pakistan, Sri Lanka and Vietnam).  (EU/UNFPA, 2006, 2007) | To improve  the sexual and reproductive health (behaviour, practices and awareness) of young  people, aged 10–24 years, including utilization of services | Before/after (no control)  Clinic and youth centre attendance data  Qualitative data | - Advocacy   - increase political and  community support for adolescent sexual and reproductive health.   - Behaviour change communication   - partnering with young people and communities to increase awareness and to improve sexual and reproductive health among adolescents and youth.   - In-school education   - e.g. teachers trained   - Peer education   - e.g. in school, in community, including life skills workshops  - e.g. bar workers trained in adolescent reproductive health and life skills   - IEC materials   - e.g. distributed in village development committees   - Youth clubs   e.g. become youth information centres and provide peer education, counselling, clinical services and vocational training   - Counselling   - e.g. telephone counselling   - Community sensitization   - e.g. sensitization workshops and support groups formed  community health, communication sessions. Parent, teacher and community leader groups set up with regular meetings, plus meetings of stakeholder group  - e.g. street theatre   - Youth-friendly services   Working to improve access to quality  youth-friendly sexual and reproductive health services  - e.g. “condom cafes” at work  - e.g. yfs at government health centres , in some cases separate male and female centres   - Institutional capacity-building   - developing the technical, planning and managerial capacity of government and local civil society organizations to meet the sexual and reproductive health needs of young people. | - Country variation in attitudes towards sexuality and sexual health   Bangladesh and Vietnam: ASRH issues are still widely considered sensitive and private issues that should not be discussed openly. Adolescents’ sexual and reproductive rights remain poorly understood. Changing these norms and involving youth actively in decision-making will take time and sustained effort.  Pakistan: The sensitivity of ASRH issues led to recognition that an effective coalition at the community level would be needed for the programme to get off the ground.   - Country variation in community support for the provision of education and services   Pakistan: Extensive community mobilization and gradual consensus among the major gatekeepers allowed the youth centres (providing services) to be established. Sensitivity surrounding the mobility of girls remained a problem and further outreach to parents was needed. The community and all the key gatekeepers are now demanding the continuation of this programme, and district politicians are ready to support it.   - Increase in the level of discussion of ASRH issues between the young and their spouses/partners, with peers, relatives including parents and with health professionals   Nepal: In general, parents and adults were reluctant to educate their children on sexual and reproductive health, fearing the sensitivity of the topic. However, in 2003–2004, the project was able to establish a supportive environment through the formation of support groups and sensitization workshops on ASRH issues. | | Qualitative data on community acceptance |
| 7. Bangladesh Frontiers programme  1999–2003  (Bhuiya et al., 2004) | To improve ASRH knowledge attitudes and behaviour of in-school and out-of-school young people | Quasi-experimental (baseline and endline population surveys (~6000 adolescents and 1500 parents) and qualitative interviews and focus groups  Test additional effect of school education  Site A  Youth-friendly services  Community  Site B  Youth-friendly services  Community  In-school education  Site C  Control | - Community   - sensitization and awareness-raising in the community (sessions with gatekeepers, parents, teachers, political and religious leaders and service providers) carried out before starting the in-school intervention;  - community events organized by peer educators;  -life skills education for adolescents  - peer education for adolescents.   - - Participatory life skills school education (led by teacher) and peer education. - Youth-friendly services   - providers trained. | - Pre-intervention FGDs found almost all gatekeepers recognized the necessity of reproductive health education and believed that in schools reproductive health information should be started from the eighth grade. Religious and community leaders believe that risk-taking behaviour will decrease if adolescents have correct reproductive health information. - Baseline survey found most parents (62-95% depending on site, with fathers slightly more in favour than mothers) approved of providing reproductive health information (including in schools) at the start of the intervention because they found it difficult for them to discuss reproductive health issues with their children. - Concluded that linking services with community resources and gaining their support for ASRH information is key to utilization of services for adolescents   - support from schools, community and clinics led to a positive enabling environment. | | - Community acceptance very high at baseline, change therefore not measured |
| 8. Senegal Frontiers programme  1999–2003  (Diop et al., 2004) | To improve ASRH knowledge, attitudes and behaviour of in-school and out-of-school urban youths | Quasi-experimental (pre-survey, post-survey, and qualitative)  Test additional effect of school education (as above) | - Community   - education sessions led by peer educators were also used to reach parents;  - sensitization and awareness-raising (parents, religious and administrative leaders attended conferences and meetings convened by the project);  - IEC agents of the Ministry of Health and Ministry of Social Affairs led discussion groups and contacted organizations such as women’s groups.   - Participatory life skills school education (led by teacher) and peer education. - Youth-friendly services   - providers trained. | - Parental approval of adolescents being able to receive services was high at the start of the intervention (all above 70%). Women’s approval was higher than men’s. The impact of the programme was unclear with approval among women increasing at both the control site (non-significant) and the intervention site without school education (significant p<0.05), but decreasing (significant p<0.05) at the other intervention site. - Community members strongly endorsed the goal of improving ASRH and this remained true through the intervention (but did not dramatically improve) although it varied according to topic. Contraception was the most sensitive topic with approval levels between 52% and 72% at endline. One significant positive change was in the site with the in-school intervention where approval of providing information about contraception increased from 53% to 66% (p<0.05). Approval of all other topics (anatomy, sexuality, early/unwanted pregnancy, STI/HIV) reached 90% or more for mothers and fathers in a ll study sites with few significant changes between surveys. - Parents traditionally lack confidence to talk openly to children about these sensitive issues but wanted to get more information so they could play a bigger role. Communication was found to improve over the course of the intervention, with adolescents more likely to seek information from adults and other qualified sources than from their friends. Nevertheless, a family atmosphere conducive to communication remained lacking, although adolescents’ expectations of more open communication had risen. - Religious leaders believed parents should discuss reproductive health openly with their children. | | - Significance tests for some changes but no confidence intervals reported - No multivariate logistic regression - Qualitative data |
| 9. Mexico Frontiers programme  1999–2003  (Vernon et al., 2004) | To improve ASRH knowledge, attitudes and behaviour of in-school and out-of-school youths | Quasi-experimental  (pre-survey, post-survey, and qualitative)  Test additional effect of school education (as above) | - Participatory life skills school education (led by teacher) and peer education. - Youth-friendly services   - providers trained.   - Community   - peer education;  - sensitization and awareness-raising (sessions with gatekeepers, parents, teachers, leaders);  - Mexfam placed a “young people coordinator” in each of the experimental group cities to train community volunteers (multipliers), and with their help disseminated sexual and reproductive health information through community events. | - Results showed that community stakeholders had quite positive attitudes at the beginning of the project regarding the delivery of information and services to adolescents. These attitudes often improved over time, but changes were observed in both the experimental and control groups so improvement cannot be linked directly to the programme. - Post-intervention surveys showed an increase in the proportion of adolescents who spoke with their mothers and fathers about reproductive health issues, with differences in characteristics of study groups controlled for. However, this change was only statistically significant (p<0.05) for communication with fathers (not mothers) and only in the control group (OR=1.667) and intervention group with in-school education (OR=1.910. Changes can therefore not be attributed to the intervention. | | - Quantitative data statistical significance to change calculated but 95% confidence intervals not reported. - Qualitative data |
| 10. Kenya Frontiers programme  1999–2003  (Askew et al., 2003) | To improve ASRH knowledge, attitudes and behaviour of in-school and out-of-school youths in rural Kenya | Quasi-experimental  (baseline and endline population surveys (~3700 (1000 boys) adolescents) and qualitative interviews  Test additional effect of school education  (as above) | - Community   - peer education;  - sensitization and awareness-raising by community development assistants and peer educators (briefing and outreach sessions with gatekeepers, parents, teachers, leaders). Religious and community leaders then went on to assist peer educators in organizing briefings and outreach events.   - - Participatory life skills school education (led by teacher) and peer education. - Youth-friendly services   - providers trained. | - Communities were very receptive to the information and dialogue about ASRH. The support and involvement of influential stakeholders (including religious leaders, teachers, young people and government representatives) was critical to increasing community discussion of ASRH and support for the program. - Parent–child communication improved significantly at the intervention site that did not include the school education intervention (p<0.05 percentage discussing SRH with at least one parent increased from 28-35%), but did not improve at the site where school education was included. It is not clear if this was due to the greater number of peer educators and community development assistants in the former, or a tendency to talk to teachers about SRH in the latter. - Surveys showed disapproval of both premarital sex and childbearing, particularly for females was high to begin with, and the interventions (especially in the intervention site without school component) reinforced these attitudes and failed to liberalise these attitudes | | - Significance testing only reported for changes in parent-child communication and OR’s and confidence intervals not included. - Qualitative data |
| ***Community participation with involvement in intervention design*** | | | | | | |
| 11. Nepal  EngenderHealth and ICRW  12–24 months of intervention (November 2000 to March 2003)  (Mathur et al., 2001) | To improve ASRH health of urban and rural young people and test the influence of youth and community participation in programming | Quasi-experimental  - baseline and endline surveys (households, adolescents, adult and service providers);  - mystery client survey;  - qualitative data collected baseline and endline. | Intervention (1)  Participatory approach  - greater youth and community participation throughout design and implementation.   - Youth-friendly services. - Peer education and counselling. - IEC campaign. - Adult peer education. - Youth clubs. - Street theatre on social norms. - Efforts to improve livelihood opportunities. - Teacher education.   Control (2 sites – 1 urban, 1 rural)  Traditional interventions   - Peer education. - Teacher training to provide education. - Youth-friendly services. | - Effects of the participatory intervention (1) were only marginally more positive on basic indicators of youth reproductive health (knowledge, attitudes and behaviour) than traditional interventions (2). However, (1) was substantially more positive in terms of the broader contextual factors such as marriage norms, empowerment, capacity-building, and sustainability. In the long term these would be expected to lead to improved reproductive health outcomes, but not in the study time. - Participatory intervention had greater effect on whether adolescents discussed reproductive health problems with their parents, this increase was greatest amongst rural males (25.6% to 51.0%) and urban females (39.4% to 72.5%) - The participatory approach was found to mobilize the community, increasing understanding of reproductive health issues and demands by adolescents and the community for information and services | | - Significance testing of adolescent behaviour change not carried out and changes in adult attitudes not reported in detail |
| 12. Burkina Faso  Advocates for Youth and Pacific Institute for Women’s Health  4–year programme 1998–2002  (Yaro et al., 2003 & 2007) | Community mobilization to help identify and tackle local priorities (includes use of services and communication regarding ASRH)  Rural adolescents | Before/after (no control)  - KAP (knowledge, attitudes and practice) survey | Community involvement in developing, implementing and evaluating the programme. Local organizations serving young people worked with community members in 20 villages to develop action plans based on local needs, including:   - Community   - awareness projects for parents, and other community members.   - Peer education   - discussions; home visits and role plays.   - Media - folk and modern. - Youth-friendly services   - providers trained; adjusted operating hours; youth area. | - Community participation in the programme was high (70% of those interviewed had participated in at least one component). The community identified infrequent use of reproductive health services and the need to improve parent–adolescent communication as priorities. - The percentage of adolescents reporting that they felt able to talk to their parents about sexuality issues rose up from 36% to 55%. - It was felt that the participatory approach helped create an enabling environment that will encourage young people to take charge of their own reproductive and sexual health, including seeking services. | | - No significance testing of behaviour changes - Anecdotal evidence |
| 13. India, Maharashtra  (ICRW and FRHS)  2001–2006  (Pande et al., 2007) | Young newly-married couples (where wife is below 22 years) with a focus on the women | Quasi-experimental  Baseline survey (1866 married girls),  mid-intervention (972 husbands in quantitative survey, 75 mothers-in-law in qualitative interviews)  Social mobilization (process evaluation)  Youth-friendly services (health worker/clinic records)  To test the relative effectiveness of addressing supply (youth-friendly services) versus demand (social mobilization) side constraints  Intervention  - 1 only social mobilization  - 1 only youth-friendly services  - 1 both  -1 control | Community (young people and others) involvement in intervention design and implementation   - Social mobilization through existing community-based organizations to address the low priority that communities place on ASRH   - strengthen youth and women’s groups (mothers-in-law and husbands drawn in to participate);  - adolescent and community education.     - Youth-friendly services (address the fact that services are not geared towards ASRH)   - improve quality and accessibility of government services;  - sensitize providers to adolescent’s needs. | - The survey of husbands showed that most husbands are now aware of basic maternal care issues, such as the need for antenatal care, and that they are willing to allow their wives to seek treatment for problems during pregnancy and childbirth. Only a minority of husbands actually accompany their wives when they seek care. - Qualitative data show that mothers-in-law, who are often the primary gatekeepers for young married women’s health seeking, are now more likely to be supportive of the young woman’s desire to seek care than they were prior to the intervention. - In general, social mobilization was more influential than the provision of youth-friendly services in improving ASRH outcomes. | | - Detailed quantitative data not reported - One limitation is that the health education sessions in the social mobilization intervention were so popular that representatives in the control arm started implementing them. This caused some contamination of the control findings. |
| 14.India, Maharashtra (ICRW and KEM)  (Pande et al., 2007) | To provide reproductive sexual health education, plus care and counselling for married adolescents (14–25 years) and to include a broad spectrum of community and family members | Quasi-experimental  Baseline survey (114 couples), process evaluation (qualitative), endline survey (74 couples) | Participatory (community involvement in design and implementation)   - Education sessions in the community. - Counselling sessions in the community   - education and counselling aimed at young women, husbands, mothers-in-law and others (community members informally participated in all activities);  - interested teachers trained as educators/counsellors;  - education and counselling components included a referral system for those requiring clinical services.   - Youth-friendly counselling and care services (provided by KEM)   - health providers trained. | - A range of community members were involved in the design of the intervention and informally participated in education activities (school teachers, health care providers, key community members etc). This feedback was considered crucial for acceptance of the activities, including referral to services. - After a period of implementation it was found that fieldworkers were more accepted and more successful at reaching young couples if they went into the community as husband-and-wife couples themselves. - Qualitative data suggests that couple communication increased where husbands and wives had previously been reluctant to discuss sexuality and reproduction with each other. - The community’s support of the intervention was clear from their request for KEM to start a programme with unmarried girls. This showed that they appreciated that girls need sexual health information before they are married. | | - Qualitative data |
